# Phosphogypsum-Modified Vinasse Shell Biochar as a Novel Low-Cost Material for High-Efficiency Fluoride Removal

**DOI:** 10.3390/molecules28227617

**Published:** 2023-11-16

**Authors:** Zheng Liu, Jingmei Zhang, Rongmei Mou

**Affiliations:** 1 School of Environmental Science and Engineering, Xiamen University of Technology, Xiamen 361024, China; 2 Fujian Engineering and Research Center of Rural Sewage Treatment and Water Safety, Xiamen 361024, China; 3 Key Laboratory of Environmental Biotechnology (XMUT), Fujian Province University, Xiamen 361024, China

**Keywords:** fluoride removal, vinasse shell, phosphogypsum, biochar, adsorption

## Abstract

In this study, vinasse shell biochar (VS) was easily modified with phosphogypsum to produce a low-cost and novel adsorbent (MVS) with excellent fluoride adsorption performance. The physicochemical features of the fabricated materials were studied in detail using SEM, EDS, BET, XRD, FTIR, and XPS techniques. The adsorption experiments demonstrated that the adsorption capacity of fluoride by MVS was greatly enhanced compared with VS, and the adsorption capacity increased with the pyrolysis temperature, dosage, and contact time. In comparison to chloride and nitrate ions, sulfate ions significantly affected adsorption capacity. The fluoride adsorption capacity increased first and then decreased with increasing pH in the range of 3–12. The fluoride adsorption could be perfectly fitted to the pseudo-second-order model. Adsorption isotherms matched Freundlich and Sips isotherm models well, giving 290.9 mg/g as the maximum adsorption capacity. Additionally, a thermodynamic analysis was indicative of spontaneous and endothermic processes. Based on characterization and experiment results, the plausible mechanism of fluoride adsorption onto MVS was proposed, mainly including electrostatic interactions, ion exchange, precipitation, and hydrogen bonds. This study showed that MVS could be used for the highly efficient removal of fluoride and was compatible with practical applications.

## 1. Introduction

The effect of fluoride in water on human health is related to its concentration. The normal growth of bones and teeth requires a certain concentration of fluoride intake (0.4 to 1.5 mg/L) [1]. However, drinking water with high levels of fluoride (above 1.5 mg/L) can cause many health problems, such as teeth mottling, skeletal fluorosis, and even changes in DNA structure [2,3]. According to the WHO, the permissible limit for fluoride in drinking water is under 1.5 mg/L [4]. In China, India, Mexico, and some African countries such as Ethiopia, Uganda, and Kenya, there are high levels of fluoride in the groundwater and surface water [5]. The recorded fluoride concentration in Lake Nakuru (Kenya) reached an alarming 2800 mg/L [6]. More than 260 million people around the world consume fluoridated water, and over 200 million people suffer from diseases associated with drinking highly fluoride-rich water, according to reports [7,8,9]. High concentrations of fluoride in water may be due to natural causes, such as the decomposition and dissolution of fluoride-containing minerals. However, the most important source is anthropogenic discharges, such as wastewater from the semiconductor industry, metallurgy, cement production, and textile dyeing [10,11]. Therefore, the removal of excess amounts of fluoride from water is an increasingly urgent issue and causes great concern.

In recent years, several defluorination methods have been explored, like adsorption, ion exchange, membrane separation, the electrochemical method, and chemical precipitation. Each of these techniques displays strengths and limitations. Ion exchange has a favorable regeneration capacity but slow treatment efficiency [12]. Membrane separation technology is highly efficient but costly [13]. The electrochemical method offers automation and reduced sludge but must tolerate rapid electrode loss [14]. Chemical precipitation has high removal efficiency but large consumption of chemical additives [15,16]. Among these treatment techniques, adsorption is widely considered by researchers and engineers for the advantages of high efficiency, extensive selectivity, and easy operation [17,18].

A mass of adsorbents has been developed for fluoride removals, such as alumina [19], activated carbon [20], fly ash [21], and various composites [22]. Among these materials, biochar is an excellent choice because of its low cost and wide range of raw materials. Nevertheless, biochar has a low adsorption capacity for anions. For this reason, it is necessary to modify biochar to enhance the performance of fluoride removal. Modification can provide significant active sites because of the changes in the surface structure of biochar and the increase of the surface functional groups [23]. Recently, several tailored biochar materials have been put forward for fluoride adsorption. The modification methods can be divided into physical and chemical modifications such as steam activation [24], impregnation [25], alkaline modification [26], and chemical reduction [27]. These explorations have presented a promising way to enhance fluoride adsorption.

Vinasse shell is the solid waste from the use of grains in the production of Chinese liquor. It is mainly used as supplementary feed for livestock and organic fertilizer. Due to the low added value, 40 million tons of vinasse shells were wasted in China in 2017 [28]. There is, therefore, a need to develop a sustainable solution to make full use of this waste. Phosphogypsum is a by-product of the phosphorus chemical industry, which cannot be easily stored and is prone to serious pollution [29,30]. Moreover, the reuse of phosphogypsum is inadequate [31]. Previous reports have shown that phosphogypsum-modified biochar can alter the surface chemistry of biochar, thereby enhancing the ability to adsorb some pollutants (phosphates, heavy metals, antibiotics, etc.) [32,33,34]. Most of these reports used co-pyrolysis of biomass and phosphogypsum to prepare adsorbent materials. They also revealed the adsorption mechanism through some physicochemical characterization and adsorption experiments. However, these studies lacked strong evidence to prove the adsorption mechanism.

In this study, phosphogypsum was used to modify vinasse shell biochar (VS), and the efficiencies of vs. and MVS in removing F^−^ from the water were compared. The specific objectives were to (1) understand the physical and chemical characteristics of the biochars using SEM, EDS, BET, XRD, FTIR, and XPS studies; (2) investigate how pyrolysis temperature, dosage, pH, and co-existing anions affect adsorption capacity; and (3) propose a mechanism for the removal of F^−^ via MVS in conjunction with experimental data and characterization analysis.

## 2. Results and Discussion

### 2.1. Characterization Analysis

#### 2.1.1. SEM-EDS

**Figure 1:**
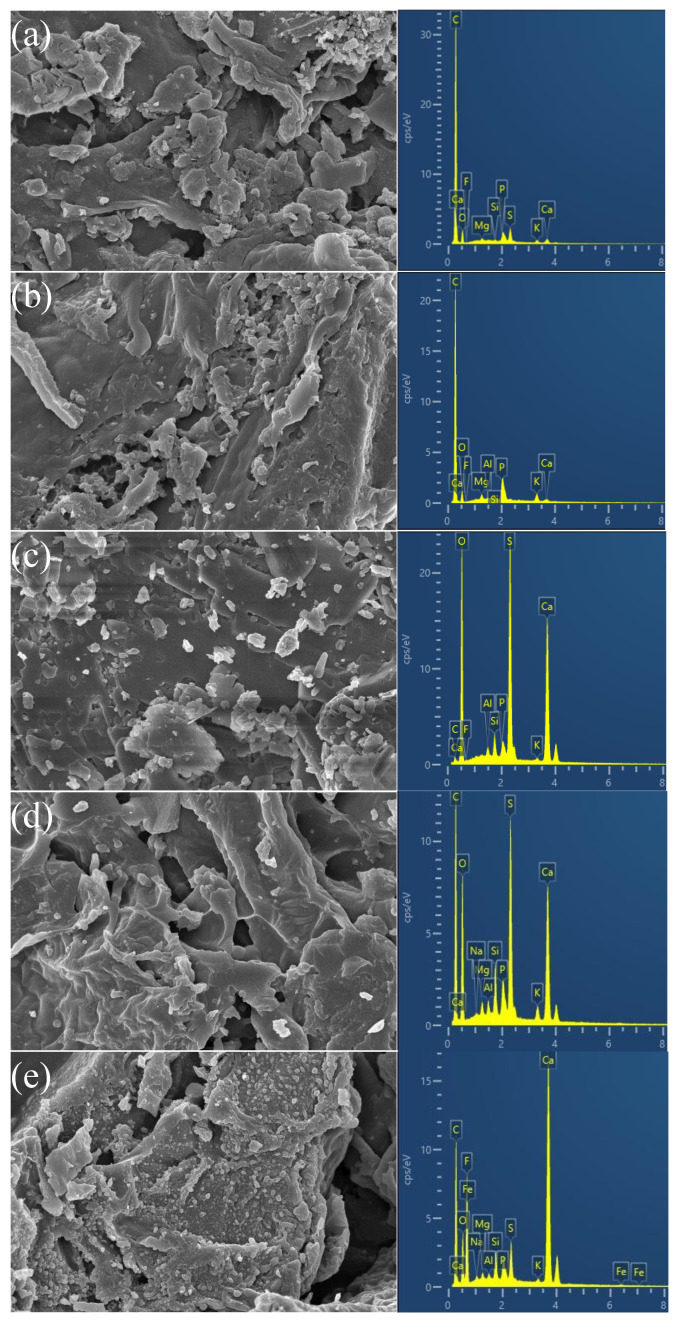
10KX SEM and EDS of (**a**) VS600; (**b**) VS600 after adsorption; (**c**) phosphogypsum; (**d**) MVS600; (**e**) MVS600 after adsorption (C_0_ = 100 mg/L; W = 0.2 g; V = 300 mL; pH = 6.0, T = 25 °C, t = 300 min).

Figure 1 shows the morphology and elemental composition of phosphogypsum, VS600, and MVS600. SEM images showed that the surface of VS600 (Figure 1a) and MVS600 (Figure 1d) had a rough appearance and some pores, whereas the phosphogypsum (Figure 1c) demonstrated a flake-like surface. It is worth noting that lots of particles appeared on the surface of MVS600 after adsorption (Figure 1e), which indicated that F^−^ was adsorbed by MVS600. EDS analysis showed that the phosphogypsum was mainly composed of Ca, S, and O. This was consistent with the fact that the main component of phosphogypsum was CaSO_4_ [35]. The EDS images also showed that MVS600 contained much more Ca, S, and O than VS600, suggesting that phosphogypsum combined well with vinasse shell biochar. After the adsorption of F^−^, there was no significant change in the fluorine content of VS600, while the content of fluorine in MVS600 raised considerably, suggesting that F^−^ was successfully adsorbed by MVS600.

#### 2.1.2. XRD

**Figure 2:**
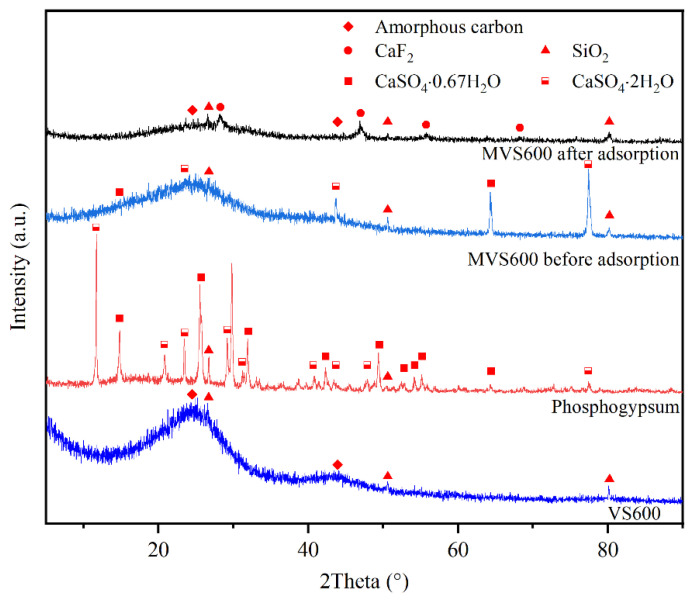
XRD patterns of VS600, phosphogypsum, and MVS600 (C_0_ = 100 mg/L; W = 0.2 g; V = 300 mL; pH = 6.0, T = 25 °C, t = 300 min).

Figure 2 shows the XRD spectra of VS600, phosphogypsum, and MVS600 before and after adsorption. The results revealed that the diffraction peaks of VS600 could be divided into amorphous carbon and SiO_2_ (PDF#46-1441). The same structures were also found in MVS600 before and after adsorption, respectively, but the peaks were less pronounced. The XRD pattern of phosphogypsum could be indexed to CaSO_4_·0.67H_2_O (PDF#47-0964), CaSO_4_·2H_2_O (PDF#33-0311), and SiO_2_. The primary elements contained in these crystals were consistent with EDS analysis. The clear, intense peaks at several sites were observed in the XRD patterns of MVS600 before adsorption. Strong signals of CaSO_4_ confirmed that the combination of phosphogypsum and vs. was successfully achieved. The most intense reflections of phosphogypsum in MVS600 were absent, probably due to the low crystallinity. MVS600 after adsorption had four CaF_2_ (PDF#35-0816) characteristic peaks at 28.3°, 47.0°, 55.8°, and 68.7°, which were attributed to (111), (220), (311), and (400) crystal faces, respectively. This suggested that F^−^ was removed in the form of CaF_2_.

#### 2.1.3. FTIR

**Figure 3:**
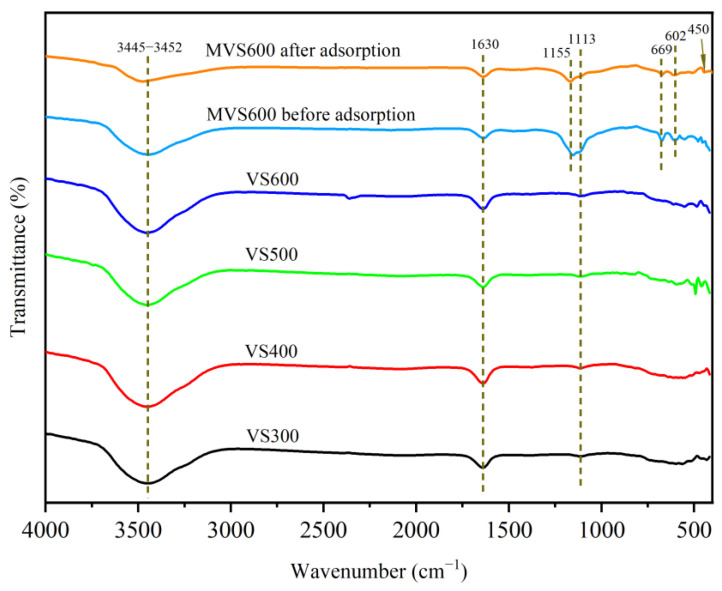
FTIR spectra of VS*x* and MVS600 (C_0_ = 100 mg/L; W = 0.2 g; V = 300 mL; pH = 6.0, T = 25 °C, t = 300 min).

The FTIR investigation was carried out on VS*x* and MVS600, and the spectra are shown in Figure 3. It showed that all biochars had similar peaks. In the FTIR spectra of all biochars, the obvious absorption bands between the wavenumbers of 3445 and 3452 cm^−1^ were due to the O–H stretching [36,37]. The absorption bands at the wavenumbers of 1630 cm^−1^ were mainly contributed by the C=C/C=O stretching [34,38]. The bands near 1113 cm^−1^ were related to the C–O stretching [39]. In the FTIR spectrum of MVS600, the clear absorption peaks at 1155, 669, and 602 cm^−1^ were related to CaSO_4_ [40,41,42], which indicated that phosphogypsum was successfully embedded in VS. The presence of CaSO_4_ confirmed that CaSO_4_ was the dominant mineral crystal in MVS600, which was indicated by the XRD analysis above. After the fluoride adsorption, the characteristic peak of –OH became weak and distorted, which was due to the ion exchange between OH^−^ and F^−^ [43,44]. Also, a slight shift in the hydroxyl peak was observed with adsorption, suggesting the possible formation of OH…F hydrogen bonds [45,46,47]. In addition, the FTIR spectrum showed that the CaSO_4_-related absorption peak was weakened, and a new peak associated with the Ca–F bond appeared at 450 cm^−1^ after fluoride adsorption [48]. This also indicated that F^−^ was removed in the form of CaF_2_.

#### 2.1.4. BET

**Figure 4:**
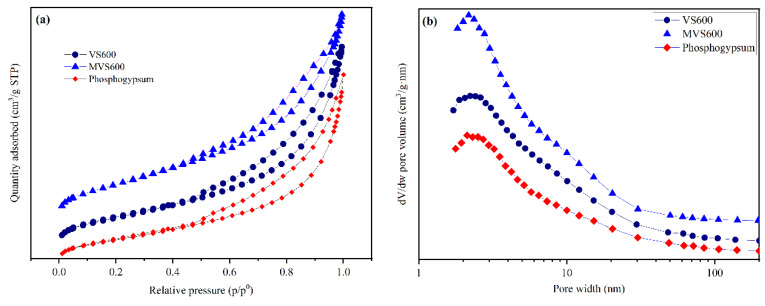
(**a**) Nitrogen adsorption–desorption curves and (**b**) pore width distributions of VS600, phosphogypsum, and MVS600.

The N_2_ adsorption–desorption curves and the corresponding pore width distributions of the materials are depicted in Figure 4. It showed that all three nitrogen adsorption–desorption isotherms belonged to type IV and had H3 hysteresis rings in terms of IUPAC. The pore width mainly ranged from 2 nm to 50 nm. This indicated that their pores had mesoporous features. The parameters related to the pore characteristics of samples are tabulated in Table 1. The BET method and NLDFT theory were adopted for the determination of the specific surface area and pore size, respectively. The average pore sizes distribution of 9.22 nm and 8.37 nm were measured from the VS600 and MVS600, further confirming the presence of abundant mesopores. The size was much larger than the size of fluoride, resulting in its easy entry into the pores [49]. VS600 and MVS600 had almost the same total pore volume. However, the BET surface area of MVS600 was larger than that of VS600, indicating that the modification improved the adsorption performance rather than reducing it.

#### 2.1.5. Zeta Potential

**Figure 5:**
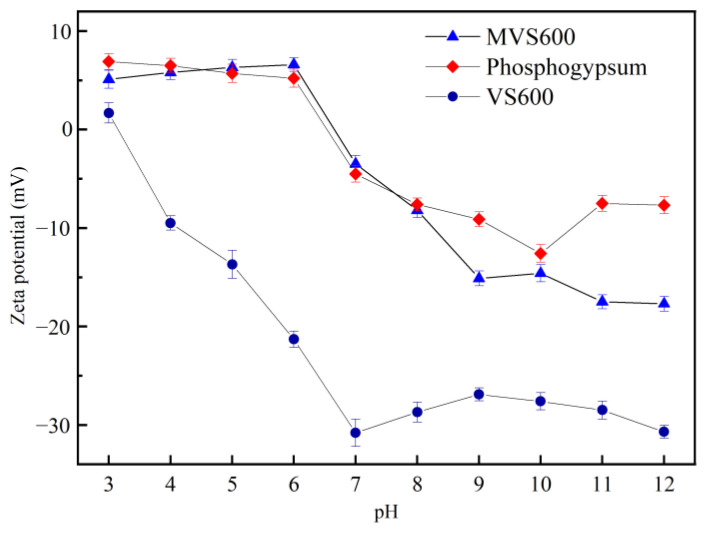
The zeta potential of VS600, phosphogypsum, and MVS600.

A possible adsorption mechanism could be inferred from the determination of the pH_pzc_. When pH_solution_ < pH_pzc_, the functional groups of the material were protonated, giving it a positively charged surface. On the contrary, the surface groups of the material would be deprotonated as pH_solution_ > pH_pzc_, so that its surface was negatively charged. As shown in Figure 5, the zeta potential of VS600, phosphogypsum, and MVS600 was determined as a function of pH. It presented that the zeta potentials of all three materials decreased with increasing solution pH. The zero charge points of VS600 and phosphogypsum were at pH 3.18 and 6.24, respectively. Compared with VS600, MVS600 had a higher zeta potential under different pH conditions. The lowest zeta potential of MVS600 was −17.70 mV, which was much higher than that of VS600 (−30.81 mV). This might be due to the strong positive charge of phosphogypsum. Therefore, electrostatic interactions might contribute to the adsorption of F^−^ anion onto MVS600. Additionally, since the electronegativity of MVS600 increased with pH, its adsorption capacity in acidic solutions was greater than that in alkaline solutions.

#### 2.1.6. XPS

**Figure 6:**
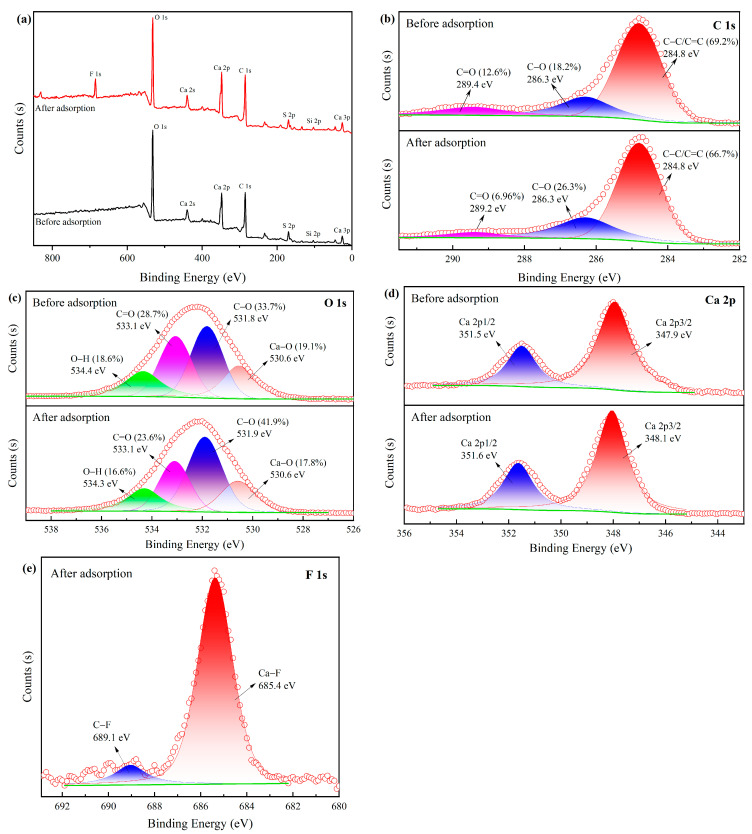
XPS analysis of MVS600: (**a**) survey scans; (**b**) C 1s; (**c**) O 1s; (**d**) Ca 2p, and (**e**) F 1s (C_0_ = 100 mg/L; W = 0.2 g; V = 300 mL; pH = 6.0, T = 25 °C, t = 300 min).

XPS analysis was performed to study the main elements and chemical valence of MVS600 before and after adsorption. Figure 6 displays the survey scans of MVS600 and the narrow-scan XPS spectra of C, O, Ca, and F. As shown in Figure 6a, MVS600 was mainly composed of C, O, Ca, S and Si. After the adsorption of fluoride, fluorine was observed in the survey spectra, which indicated that F^−^ was successfully adsorbed by MVS600. These results were consistent with the EDS analysis. Figure 6b shows that C1s spectra could be described as three peaks, namely C–C/C=C, C–O, and C=O [50,51]. After adsorption, the peak of C=O shifted from 289.4 eV to 289.2 eV, and the content also decreased, suggesting that C=O groups were associated with fluoride adsorption. The XPS spectra of O1s in Figure 6c could be assigned to four different components: Ca–O, C–O, C=O, and O–H. After adsorption, the content of Ca–O decreased from 19.1 to 17.8%, implying that Ca contributed to the removal of fluoride. Additionally, the area ratio of the O–H peak decreased from 18.6 to 16.6%, which was attributed to the presence of ligand exchange between hydroxyl and fluorine ions. The Ca2p XPS spectrum of MVS600 consisted of two peaks at 351.5 and 347.9 eV, as shown in Figure 6d. Their binding energies underwent a small change after adsorption, indicating that CaSO_4_ was an important factor in helping to remove fluoride. As shown in Figure 6e, the F1s XPS spectra decomposed into two peaks at 685.4 and 689.1 eV for Ca–F and C–F bonds, respectively. The new observation of the C–F could be the substitution of hydroxyl groups on the surface of MVS600 by F^−^. The binding energy of the new Ca–F peak was higher than that of the NaF peak (684.5 eV), indicating the formation of CaF_2_. These results confirmed that the removal of F^−^-contained chemical processes.

### 2.2. Batch Adsorption

#### 2.2.1. Effect of Pyrolysis Temperature

**Figure 7:**
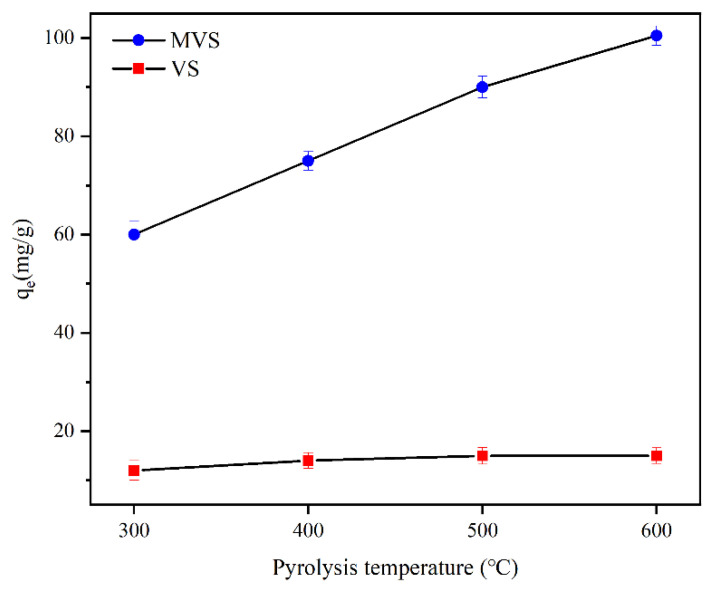
The adsorption capacities of fluoride by biochars at different pyrolysis temperatures (C_0_ = 100 mg/L; V = 300 mL; W = 0.2 g; pH = 6.0; T = 25 °C, t = 300 min).

The physical and chemical properties of the biochar had an impact on the adsorption performance of biochar and were influenced by the pyrolysis temperature [52]. Figure 7 illustrates the fluoride adsorption capacities by biochars at different pyrolysis temperatures. MVS exhibited higher fluoride adsorption than vs. at all different pyrolysis temperatures. When the pyrolysis temperature reached 600 °C, the adsorption capacity of fluoride by MVS600 amounted to 100.5 mg/g, which was much larger than that by VS600. This proved that the phosphogypsum modification contributed to the increase of fluoride adsorption. Figure 7 also shows that the adsorption capacity of fluoride rose with increasing pyrolysis temperature. However, the increase by MVS*x* was more pronounced, while the trend of increase by VS*x* was much less apparent. This might be because pyrolysis temperature positively affected the textural and adsorptive properties of VS*x* biochar. As the pyrolysis temperature rose, the material inside VS*x* was decomposed and released, and the number of pores was increased [53]. More abundant pores, the increase in surface area, and the change in the surface chemical property provided greater adsorption possibilities and more adsorption sites, which were helpful for the adsorption of fluoride [54,55,56].

#### 2.2.2. Effect of Dosage

**Figure 8:**
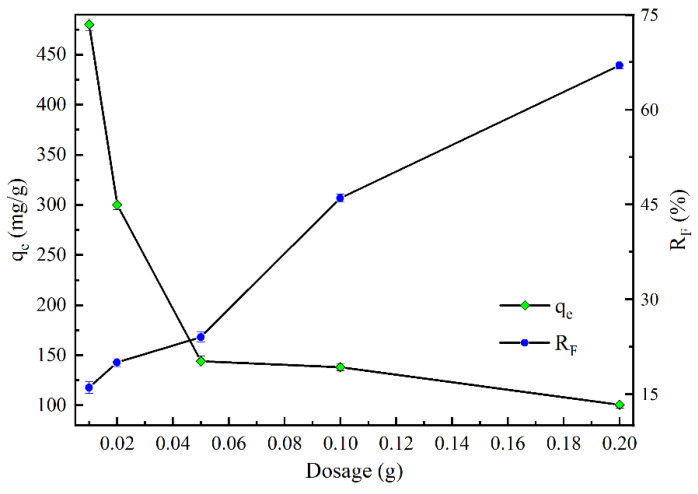
Adsorption capacity and removal efficiency of fluoride by MVS600 at different dosages (C_0_ = 100 mg/L; V = 300 mL; pH = 6.0; T = 25 °C, t = 300 min).

Figure 8 presents the influence of the dosage of MVS600 on the fluoride adsorption capacity and removal efficiency. It was noticed that as the dosage rose from 0.01 g to 0.2 g, the adsorption capacity gradually declined from 480 mg/g to 100.5 mg/g. Similar findings were also obtained for fluoride adsorption by PAA@C-CS and modified chitosan [57,58]. This was due to the constant initial concentration of fluoride. Meanwhile, particle agglomeration was more likely to occur when more adsorbent was added to a given volume of solution, reducing the adsorption active sites per unit mass. However, the removal efficiency (*R*_F_ = (1 − *C*_e_/*C*_0_) × 100%) gradually increased from 16 to 67% with the increase of dosage from 0.01 g to 0.2 g. This was because the increase in the dosage of MVS600 provided more adsorption sites.

#### 2.2.3. Effect of pH

**Figure 9:**
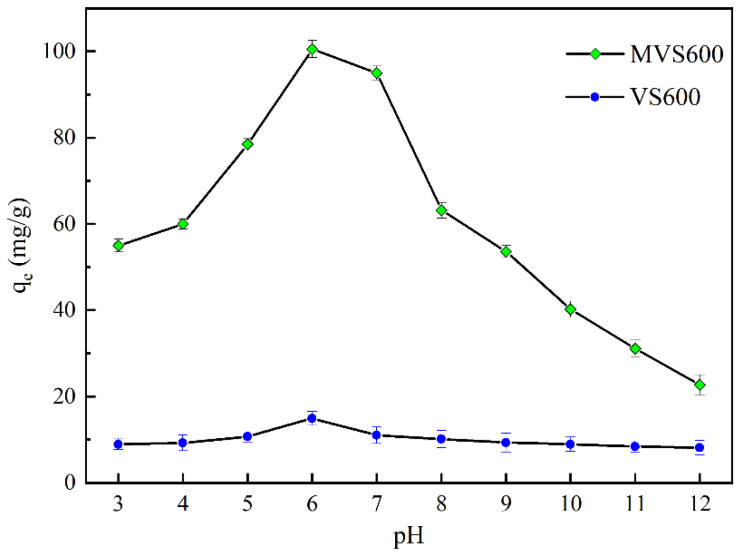
Adsorption capacity of fluoride by MVS600 at different pHs (C_0_ = 100 mg/L; W = 0.2 g; V = 300 mL; T = 25 °C, t = 300 min).

Previous reports indicated that the solution pH had a big influence on fluoride adsorption [59]. Figure 9 presents how pH affected fluoride adsorption. The maximum adsorption capacity occurred around pH = 6 for both MVS600 and VS600. However, the adsorption of fluoride by MVS600 was more influenced by pH. The results also displayed that the fluoride adsorption by both MVS600 and VS600 increased firstly and then decreased with increasing pH in the range of 3−12. Under acidic pH conditions, more free H^+^ ions protonated the MVS600 surface, thus contributing to the adsorption of the anion F^−^ onto MVS600. The protonation also dehydrated the residual cellulose (if any remained on the surface of the material) and produced electrophilic carbon, which was electrostatically attracted to F^−^ [60]. However, the form of F^−^ in the aqueous solution varied with the pH. When pH < 3.18, F^−^ was mostly present as HF molecules [61]. HF was more difficult to be adsorbed than F^−^ [62]. Therefore, at low pH, the fluoride adsorption by MVS600 and VS600 was lower. At alkaline pH, more free OH^−^ ions deprotonated the MVS600 surface and made it negatively charged. This was not conducive to the adsorption of F^−^ anion. Thus, the fluoride adsorption was significantly lower at basic pH. The previous study also revealed that OH^−^ in alkaline solutions strongly interfered with the adsorption of fluoride [63]. This suggested that electrostatic forces were mainly responsible for the influence of pH on fluoride adsorption.

#### 2.2.4. Effect of Co-Existing Anions

**Figure 10:**
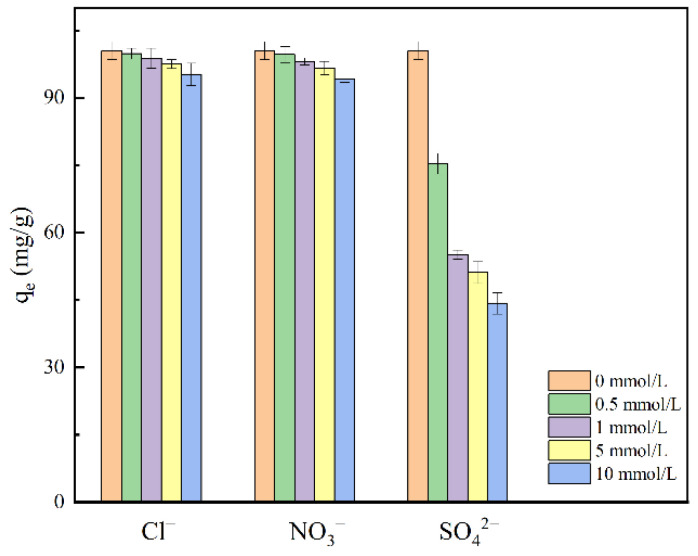
Adsorption capacity of fluoride by MVS600 at different dosages (C_0_ = 100 mg/L; W = 0.2 g; V = 300 mL; pH = 6.0; T = 25 °C, t = 300 min).

The co-existing anions in the water may be in competition with F^−^ for active adsorption sites, thus suppressing the uptake of fluoride ions. Figure 10 shows that when the Cl^−^ concentration was raised from 0 to 10 mmol/L, the fluoride adsorption capacity gradually decreased from 100.5 mg/g to 95.2 mg/g. When the NO_3_^−^ concentration increased from 0 to 10 mmol/L, the fluoride adsorption capacity gradually decreased from 100.5 mg/g to 94.3 mg/g. In contrast, SO_4_^2−^ significantly affected the adsorption capacity of fluoride, showing the strongest interference. When SO_4_^2−^ concentration increased from 0 to 10 mmol/L, the fluoride adsorption capacity was reduced by 56.0%. Cl^−^ and NO_3_^−^ have a low affinity, and their adsorption is usually a weak binding to the active site. While SO_4_^2−^ can form a strong binding with the adsorbent through a complex of outer and inner spheres [64]. At the same time, SO_4_^2−^ consumed Ca^2+^ on the outer surface of MVS600 (Ca^2+^ + SO_4_^2−^ → CaSO_4_, K_sp_ = 9.1 × 10^−6^), further inhibiting the fluoride adsorption. Therefore, the influence of chloride and nitrate in the wastewater could be neglected in practical applications of fluoride adsorption.

### 2.3. Adsorption Kinetics Analysis

**Figure 11:**
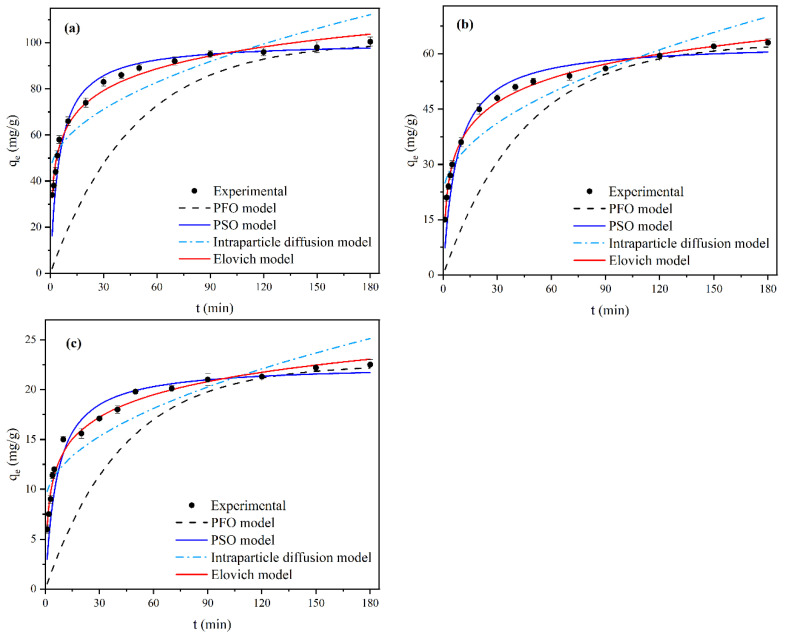
The fitted curves of the kinetic models: (**a**) C_0_ = 100 mg/L, (**b**) C_0_ = 60 mg/L, (**c**) C_0_ = 20 mg/L (W = 0.2 g; V = 300 mL; pH = 6.0; T = 25 °C).

Adsorption kinetics analysis was crucial to explore the adsorption mechanism. As shown in Figure 11, for different F^−^ initial concentrations, the fluoride adsorption capacity of MVS600 increased quickly during the first 30 min, then slowed down and reached equilibrium after about 180 min. This was because there was a large concentration gradient at the solid–liquid interface in the initial phase, and the surface of MVS600 could provide sufficient adsorption active sites. With increasing contact time, the active sites on the MVS600 surface and the macropore adsorption decreased. At this moment, the micropore adsorption became the dominant mode, resulting in a slow increase in fluoride adsorption. Finally, the surface sites on the MVS600 were gradually covered, and the adsorption reached dynamic equilibrium.

To better understand the adsorption behavior of F^−^ on MVS600, four frequently used kinetic models were adopted to assess their suitability for the kinetics data. The fits were in non-linear modes. Figure 11 and Table 2 show the fitted curves of the kinetic models and the corresponding model constants, respectively. From the low correlation coefficient *R*^2^, as well as the high deviation between calculated values *q*_e,cal_ and experimental value *q*_e,exp_, it could be concluded that the PFO model and the intraparticle diffusion model were not compatible well with the kinetic data. In comparison, the PSO model showed comparatively high *R*^2^ (>0.99) and lower standard deviation (SD) at all three concentrations, and *q*_e,cal_ was also closer to the *q*_e,exp_. Thus, the PSO model better matched the solid–liquid adsorption kinetics. This implied that fluoride adsorption on MVS600 was a second-order reaction at a low adsorbate-to-adsorbent ratio and that chemisorption was possibly involved in the adsorption process [65].

### 2.4. Adsorption Isotherm Study

Isotherms study was usually used to investigate the way in which the adsorbate adhered to the surface of the adsorbent [66]. In this work, four widely-used isotherm models were adopted to explain what happened in the adsorption of F^−^ on MVS600, including Langmuir, Freundlich, Temkin, and Sips models. The Temkin isotherm reflects the distribution of the binding energies during the adsorption process. The Sips model is a hybrid model that incorporates characteristics of both the Langmuir and Freundlich models [67].

**Figure 12:**
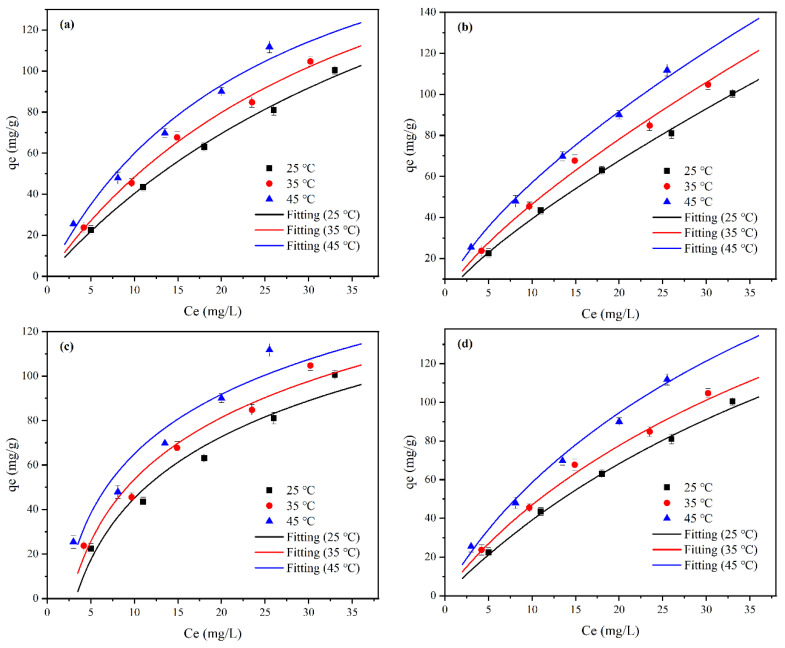
The fitted curves of isotherm models: (**a**) Langmuir; (**b**) Freundlich; (**c**) Temkin; and (**d**) Sips (C_0_ = 20, 40, 60, 80, 100 mg/L; W = 0.2 g; V = 300 mL; pH = 6.0, t = 300 min).

According to the non-linear equations, the isothermal fitting plots at 25 °C, 35 °C, and 45 °C have been drawn in Figure 12. Table 3 lists the corresponding parameters. A maximum adsorption capacity of 111.8 mg/g was obtained at 45 °C and C_0_ = 100 mg/L. It clearly demonstrated that the equilibrium adsorption capacity raised with increasing initial concentration at a certain temperature. This phenomenon might be due to the fact that at a low concentration, lots of active sites on the surface of MVS600 were not yet occupied, thus providing an opportunity for more F^−^ to be adsorbed by MVS600. Simultaneously, the high concentration enhanced the mass transfer driving force at the solid–liquid interface, which also contributed to the increase in adsorption capacity. It was also noticed that the equilibrium adsorption capacity increased as the solution temperature rose, suggesting that the adsorption was an endothermic process.

On the basis of the correlation coefficient *R*^2^, it was observed that all four isotherm models were in very good agreement with the adsorption results. However, at all three temperatures, the Freundlich and Sips isotherm models fitted the experimental data better than the Langmuir and Temkin models due to higher *R*^2^ (>0.99) and lower standard deviation (SD). The values of the Freundlich constant 1/*n* and the Sips constant *m*_s_ offered a sign of the ease of adsorption. It was widely accepted that values of 1/*n* and *m*_s_ in the range of 0–1 indicated favorable adsorption features [68,69]. Table 3 shows that the constant 1/*n* at different temperatures was 0.6844–0.7829, which mainly represented that the chemisorption happened at the time of the adsorption of F^−^ onto MVS600 [70]. This inference was in accordance with the results of the kinetics analysis. From the Sips isotherm model, it could be deduced that the F^−^ adsorption onto MVS600 might be multi-layer adsorption on a heterogeneous interface, which was involved in diversiform mechanisms. Furthermore, on the basis of the Sips isotherm model, MVS600 achieved the maximal adsorption capacity of 290.9 mg/g at 25 °C.

Compared to most of the reported materials (Table 4), the F^−^-adsorption capacity of MVS600 was far greater. Accordingly, the MVS600 could be considered a good alternative for the removal of fluorine contamination from the aqueous solution.

### 2.5. Adsorption Thermodynamic Study

**Figure 13:**
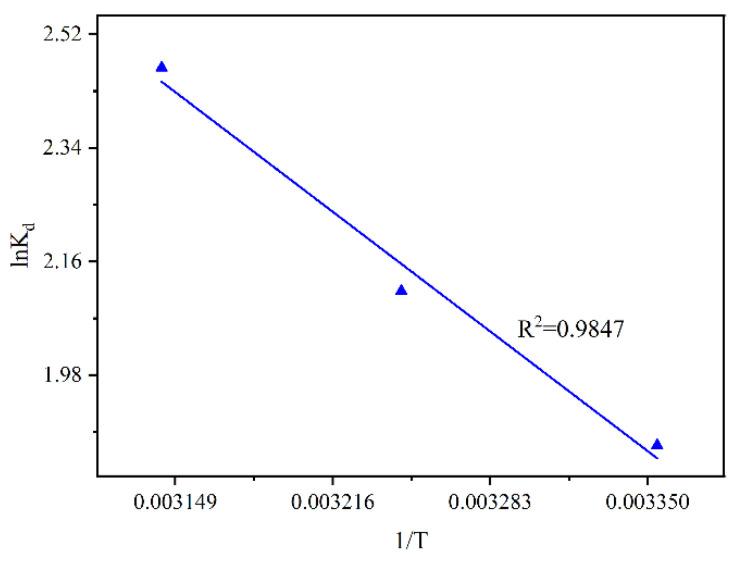
Thermodynamic fitting plot analyzed using the Van’t Hoff equation (C_0_ = 100 mg/L; W = 0.2 g; V = 300 mL; pH = 6.0, t = 300 min).

The thermodynamic study was used for the description of the thermal effects of the adsorption reaction. The Freundich isothermal model described the experimental data well; therefore, the *K*_d_ of the model was used to calculate the thermodynamic parameters. Figure 13 displays the fitting plot analyzed using the Van’t Hoff equation. Based on the slope and intercept, the thermodynamic parameters determined at 25 °C, 35 °C, and 45 °C are tabulated in Table 5. The fitting plot showed a good linearity (*R*^2^ = 0.9847). Table 5 shows that the equilibrium constant *K*_d_ increased with temperature, and it was concluded that higher temperatures were beneficial to the adsorption. Δ*G*^0^ < 0 suggested that F^−^ adsorption on MVS600 was favorable and spontaneous [82]. It should be noted that both Δ*H*^0^ and Δ*S*^0^ were positive, from which it could be inferred that they had distinct effects on the adsorption behavior. Δ*H*^0^ > 0 meant that the adsorption took place endothermically between 25 °C and 45 °C, and energy would be consumed when F^−^ was transferred from the solution onto the surface of MVS600. This result was in accordance with the conclusion of the isothermal analysis. The same conclusion was also found in other fluoride adsorption experiments [70,83,84]. In fact, high temperatures promoted an increase in the kinetic energy of the adsorbent molecules, thereby accelerating heat and mass transfer. Furthermore, more adsorption sites on the adsorbent were activated by the increase in temperature [85].

The average activation energy *E*_a_ of 26.09 kJ/mol also reflected the endothermic nature of the interaction between F^−^ and MVS600 [86]. The positive Δ*S*^0^ of the adsorption indicated that some structural changes occurred in the adsorption process, and more randomness appeared in the adsorbent-solution interface. This was probably ascribed to the reduction of the hydrated ions and the release of H_2_O on the surface of MVS600 [87].

### 2.6. Mechanism of F^−^ Adsorption

**Figure 14:**
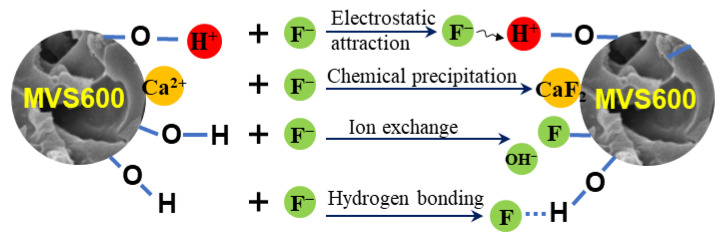
The schematic diagram for the mechanism of F^−^ adsorption.

Based on the experimental data and characterization, the mechanisms for the F^−^ adsorption with the MVS600 were summarized as electrostatic interactions, ion exchange, precipitation, and hydrogen bond (Figure 14).

(1) Ion exchange. The analysis of FTIR and XPS revealed that the surface of MVS600 was rich in hydroxyl groups, and the content of the hydroxyl group became less after adsorption. This indicated that when fluoride approached MVS600, the hydroxyl groups on the surface were replaced by fluorine ions, as shown in Equation (1);
(2) Chemical precipitation. Both XRD analysis and XPS analysis confirmed the production of CaF_2_. F^−^ and Ca^2+^ on the MVS600 surface had a very tight attraction for each other and easily formed CaF_2_ precipitates, as shown in Equation (2);
(3) Electrostatic attraction. It could be seen from the zeta potential analysis under low pH conditions that the C–O groups on the surface of MVS600 were protonated to C–OH^+^ and carried a large number of positive charges. Negatively charged fluorine ions were removed by electrostatic interaction with them, as shown in Equation (3). The formation of weakly ionized HF under strongly acidic conditions and deprotonation of surface functional groups under alkaline conditions reduced the degree of F adsorption. Under near-neutral conditions, F^−^ adsorption could be described using other equations;
(4) Hydrogen bonding. Due to the high electronegativity of O and F, the shared electron pair of –OH groups on the surface of MVS600 was biased toward O, so H easily interacted with F and formed an –OH⋯F hydrogen bond, as shown in Equation (4). This might be responsible for the change in O–H binding energy before and after adsorption in the XPS spectra.

MVS600−*Surface*−OH + F^−^ → MVS600−*Surface*−F + OH^−^ (1)

MVS600−*Surface*−Ca^2+^ + F^−^ → MVS600−*Surface*−CaF_2_ *K*_sp_ = 3.9 × 10^−11^ (2)

MVS600−*Surface*−O + H_3_O^+^ + F^−^ → MVS600−*Surface*−OH^+^−F^−^ + H_2_O (3)

MVS600−*Surface*−O−H → MVS600−*Surface*−O−H⋯F^−^ (4)

## 3. Experimental Section

### 3.1. Reagents

All chemicals with analytical grade were obtained from Sinopharm Co., Ltd., Beijing, China. The stock solution of F^−^ was prepared with sodium fluoride (NaF). The concentration of F^−^ was measured using an F^−^ selective electrode (Bante 931). Before the measurement, a volume of TISAB Ⅱ solution was added to the F^−^-containing sample. The solution preparation and dilution were carried out using deionized water. The pHs of the F^−^-containing solution were adjusted with hydrochloric acid and sodium hydroxide.

### 3.2. Preparation of Materials

Vinasse shells and phosphogypsum (CaSO_4_ ≥ 75% *w*/*w*) were purchased on the internet. The shells were first rinsed with water to wash off debris and dried with phosphogypsum at 105 °C to constant weight. After that, shells and phosphogypsum were, respectively, grounded to powders with 0.18–0.425 mm particle size. Next, phosphogypsum powder, vinasse shells powder, and deionized water were mixed in a weight ratio 1:2:3 and stirred for 30 min. Then, the mixture was dried in a drying machine at 105 °C to constant weight. Finally, the dried mixing was heated for 1 h at 10 °C/min in a nitrogen-filled tube furnace. The product obtained was MVS, referred to as MVS*x*, where *x* represented the pyrolysis temperature (*x* = 300, 400, 500, and 600 °C). Unmodified vinasse shells were also treated with the same conditions to obtain biochar labeled as VS*x*.

### 3.3. Physicochemical Characterization

The microstructure and containing elements of the materials were analyzed using SEM (Zeiss Ultra Plus, Carl Zeiss AG, Oberkochen, Germany) and EDS (Oxford X-max 50, Oxford Instruments, Oxford, UK). The phase composition was examined using XRD (Bruker D8 Advance, Bruker Co., Saarbrücken, Germany) using Cu-Kα radiation. The chemical groups of the material were investigated using FTIR (Nicolet 380, Thermo Fisher Scientific Inc., Carlsbad, USA) with a wavenumber range of 4000–400 cm^−1^. The specific surface area and pore parameters of the materials were acquired using N_2_ adsorption at −196 °C on a physisorption instrument (TriStar II 3020, Micromeritics Instrument Co., Norcross, GA, USA). The zeta potentials of the materials were determined using a potentiometric analyzer (Malvern Zetasizer Nano ZS90, Malvern Panalytical Ltd., Malvern, UK). The surface chemistry of the materials before and after adsorption was acquired using a photoelectron spectrometer (Thermo Scientific EscaLab 250Xi, Thermo Fisher Scientific Inc., Carlsbad, CA, USA) with a monochromatized Al Kα X-ray source.

### 3.4. Adsorptive Experiments

The adsorptive experiments were conducted using polypropylene conical beakers to observe the adsorption performance of materials. A certain mass of VS*x* or MVS*x* was added to the F^−^ solution containing a fixed initial concentration (*C*_0_, mg/L) and oscillated at 200 r/min in a thermotank at 25 °C for 300 min. Using a 0.45-μm PES filter, the suspension was filtered after sampling. The effect of pyrolysis temperature (300, 400, 500, and 600 °C), dosage (0.01, 0.02, 0.05, 0.1, and 0.2 g), and solution pH (3–12) on the adsorption performance of F^−^ was examined. Detailed experimental conditions were described in the illustrations. To evaluate the impact of anions on fluoride adsorption, NaCl, NaNO_3,_ and Na_2_SO_4_ were, respectively, added to the solution at pH = 6.0, and the concentrations of the anions were set to 0.5, 1, 5, and 10 mmol/L. Each group of experiments was repeated thrice to get the average adsorption capacity. The following equations were used to obtain the F^−^ adsorption capacities.

(5) $$q_{e}=\frac{(C_{0}-C_{e})\times V}{W}\;$$

(6) $$q_{t}=\frac{(C_{0}-C_{t})\times V}{W}\;$$

where *q*_e_ (mg/g) was the equilibrium adsorption capacity, *q*_t_ (mg/g) was the F^−^ adsorption capacity at time *t*, *V* (L) represented the volume of the suspension, *W* (g) represented the dosage of the material, *C*_t_ (mg/L) represented the concentration at time *t*, and *C*_e_ (mg/L) referred to the F^−^ concentration at equilibrium.

Batch experiments were also performed to determine the adsorption mechanisms, namely adsorption kinetics, isotherms, and thermodynamics. The solution temperatures were set at 25, 35, and 45 °C to study the thermodynamics. The most commonly used models were utilized to fit the adsorption results.

#### 3.4.1. Adsorption Kinetics Modeling

Adsorption kinetics modeling was helpful for further studying the rate-limiting steps and adsorption rates. The pseudo-first-order (PFO) model, pseudo-second-order (PSO) model, intraparticle diffusion model, and Elovich model were employed to model the experiment data, which could be described by Equations (7)–(10), respectively.

(7) $$q_{t}=q_{e}\left(1-e^{-k_{1}t}\right)\;$$

(8) $$q_{t}=\frac{k_{2}q_{e}^{2}t}{{1+k}_{2}q_{e}t}\;$$

(9) $$q_{t}=k_{3}t^{0.5}+C_{i}$$

(10) $$q_{t}=\frac{1}{\beta }\ln\alpha \beta +\frac{1}{\beta }lnt$$

where *k*_1_ (1/min), *k*_2_ (g/(mg·min)), *k*_3_ (mg/(g·min^0.5^)), and *α* (mg/(g·min)) denoted the rate constants of the corresponding models; *C*_i_ (mg/g) and *β* (g/mg) referred to the model constants.

#### 3.4.2. Adsorption Isotherm Modeling

Adsorption isotherm results were modeled by the non-linear equations, that is the Langmuir model, the Freundlich model, the Temkin model, and the Sips model. The formulas could be given by the equations below, respectively [88].

(11) $$q_{e}=\frac{q_{m}K_{L}C_{e}}{1+K_{L}C_{e}}$$

(12) $$q_{e}=K_{F}C_{e}^{1/n}\;$$

(13) $$q_{e}=A_{T}\ln(B_{T}C_{e})\;\;$$

(14) $$q_{e}=\frac{q_{m}{\left(K_{S}C_{e}\right)}^{m_{S}}}{1+{\left(K_{S}C_{e}\right)}^{m_{S}}}\;$$

where *q*_m_ (mg/g) represented the maximum adsorption capacity; *K*_L_ (L/mg) and *K*_F_ ((mg/g) × (L/mg)^1/n^) were the Langmuir and Freundlich constants, respectively; *n* denoted the Freundlich constant related to adsorption intensity; *A*_T_ and *B*_T_ (L/mg) denoted Temkin constants; *K*_s_ was related to the adsorption energy, and *m*_s_ denoted the Sips model exponent index related to adsorption heterogeneity.

#### 3.4.3. Adsorption Thermodynamics

The thermodynamic study could be used to ascertain the direction and driving force of adsorption reactions. The related parameters, namely Gibb’s free energy Δ*G*^0^, enthalpy Δ*H*^0^, and entropy Δ*S*^0^, could be estimated based on Equations (15) and (16).

(15) $$\Delta G^{0}=-RTlnK_{d}$$

(16) $$lnK_{d}=-\frac{\Delta H^{0}}{R}\frac{1}{T}+\frac{\Delta S^{0}}{R}$$

Here, *R* denoted the ideal gas constant, *T* (K) denoted the reaction temperature, and *K*_d_ denoted the thermodynamic distribution coefficient.

Based on the above parameters, the average activation energy *E*_a_ of the adsorption could be determined by Equation (17) [89].

(17) $$E_{a}=\Delta H^{0}+RT\;$$

## 4. Conclusions

Phosphogypsum was used to modify vinasse shell biochar for the removal of fluoride from aqueous solutions. The modified material (MVS600) gave excellent fluoride adsorption performance with an adsorption capacity of 290.9 mg/g according to adsorption isotherms experiments. Kinetics investigations showed that the fluoride adsorbed onto MVS600 followed the PSO model. Spontaneous and endothermic adsorption was shown by thermodynamic data. Mechanistically, the removal of fluoride by MVS600 could be attributed to electrostatic interactions, ion exchange, precipitation, and hydrogen bonds. Because of its low cost and superior capacity, MVS600 was a good candidate for dealing with high levels of fluorine contamination or for long-term use. The outcomes of this research were beneficial to the treatment of fluoride-polluted water as well as the resourceful use of vinasse shells and phosphogypsum.

## Figures and Tables

**Table 1:** Textural properties of VS600 and MVS600.

Samples	BET Surface Area (m^2^/g)	Total Pore Volume (cm^3^/g)	Average Pore Diameter (nm)
VS600	64.5	0.1487	9.22
MVS600	71.3	0.1492	8.37

**Table 2:** Adsorption kinetic model constants.

C_0_ (mg/L)	Pseudo-First Order Model				
	q_e,exp_ (mg/g)	q_e,cal_ (mg/g)	k_1_ (1/min)	R^2^	SD
100	100.5	46.99	0.02154	0.9391	0.264
60	63.0	37.41	0.02222	0.9583	0.223
20	22.5	12.38	0.02347	0.9606	0.229
	**Pseudo-Second Order Model**				
	**q_e,exp_ (mg/g)**	**q_e,cal_ (mg/g)**	**k_2_ (g/mg·min)**	**R^2^**	**SD**
100	100.5	101.3	0.001929	0.9989	0.0192
60	63.0	63.86	0.002108	0.9967	0.0526
20	22.5	22.84	0.006834	0.9977	0.122
	**Intraparticle Diffusion Model**				
	**q_e,exp_ (mg/g)**	**C (mg/g)**	**k_3_ (mg/g·min^1/2^)**	**R^2^**	**SD**
100	100.5	42.84	5.17	0.8510	9.01
60	63.0	21.28	3.635	0.8882	5.37
20	22.5	8.53	1.237	0.8643	2.04
	**Elovich Model**				
	**q_e,exp_ (mg/g)**	**α (mg/(g·min))**	**β (g/mg)**	**R^2^**	**SD**
100	100.5	150.9	0.07321	0.9862	2.74
60	63.0	44.76	0.1058	0.9963	0.973
20	22.5	21.96	0.3082	0.9867	0.638

**Table 3:** Parameters of adsorption isotherm model at different temperatures.

Isotherm Models	Parameters	Values (25 °C)	Values (35 °C)	Values (45 °C)
Langmuir	K_L_/(L/mg)	0.01887	0.02678	0.04010
	q_m_/(mg/g)	253.8	228.8	209.2
	R^2^	0.9654	0.9419	0.9136
	SD	2.14	2.64	4.47
Freundich	K_F_ ((mg/g) × (L/mg)^1/*n*^)	6.482	8.276	11.79
	1/n	0.7829	0.7493	0.6844
	R^2^	0.9988	0.9940	0.9980
	SD	1.26	3.14	2.11
Temkin	A_T_/(L/mg)	39.91	40.15	38.66
	B_T_	0.3091	0.3792	0.5371
	R^2^	0.9598	0.9677	0.9366
	SD	6.14	5.73	8.55
Sips	q_m_	290.9	321.9	353.5
	K_s_	0.015	0.014	0.016
	m_s_	0.9823	0.9002	0.8836
	R^2^	0.9963	0.9937	0.9925
	SD	1.83	2.59	3.12

**Table 4:** The adsorption capacity of fluoride by different reported materials.

Material	Q_max_ (mg/g)	Conditions	Reference
Zirconium impregnated carbon	40.02	W = 10 g/L, 4.0, 25 °C	[71]
SWCNTs	63.2	W = 0.6 g/L, pH = 6.0, T = 30 °C	[72]
GAC-Fe_3_O4	2.74	W = 1 g/L, pH = 3.0, T = 25 °C	[73]
*Mytilus coruscus* shells	82.93	W = 3.33 g/L, pH = 7.0, T = 25 °C	[74]
Y-Zr-Al composite	31.0	W = 1 g/L, pH = 7.0, T = 25 °C	[75]
Al_2_O_3_ microfiber clusters	14.96	W = not mentioned, pH = 5.0, T = 40 °C	[76]
AC-Si-Mg-La	54.83	W = 0.2 g/L, pH = 5.0, T = 25 °C	[17]
La/Fe/Al loaded rice straw biochar	111.11	W = 1 g/L, pH = 7.0, T = 25 °C	[77]
DTAB/H_2_O_2_–clay	53.66	W = 2 g/L, pH = 2.0, T = 25 °C	[78]
HAO@GO	129.23	W = 0.05 g/L, pH = 7.0, T = 25 °C	[79]
rGO-Ce/Ag	434.78	W = not mentioned, pH = 7.0, T = 25 °C	[80]
Ca modified Mg-Zr MMOs	370.37	W = 0.5 g/L, pH = 7.0, T = 25 °C	[81]
MVS600	290.9	W = 0.667 g/L, pH = 7.0, T = 25 °C	Present study

**Table 5:** Thermodynamic parameters at 25 °C, 35 °C and 45 °C.

T (°C)	K_d_	ΔG^0^ (kJ/mol)	ΔH^0^ (kJ/mol)	ΔS^0^ (J/(mol∙K))
25	6.482	−4.633	23.53	94.29
35	8.276	−5.414	23.53	94.29
45	11.79	−6.526	23.53	94.29

## Data Availability

Data are contained within the article.
